# Efficacies of preoperative prism adaptation test and monocular occlusion for detecting the maximum angle of deviation in intermittent exotropia

**DOI:** 10.1186/s12886-021-02060-9

**Published:** 2021-08-21

**Authors:** Ryota Takada, Fumiko Matsumoto, Akemi Wakayama, Takuya Numata, Fumi Tanabe, Kosuke Abe, Shunji Kusaka

**Affiliations:** Department of Ophthalmology, Kindai University Faculty of Medicine, 377-2 Ohnohigashi, Osaka-Sayama, Osaka 589-8511 Japan

**Keywords:** Intermittent exotropia, Prism adaptation test, Monocular occlusion, Maximum angle of deviation

## Abstract

**Background:**

The efficacies of prism adaptation test (PAT) and monocular occlusion (MO) and their optimal test durations to detect the maximum angles of deviation at near and distance in eyes with intermittent exotropia (IXT) were assessed and compared.

**Methods:**

We retrospectively reviewed the medical records of 72 patients with IXT. All the patients had undergone the initial strabismus surgery between April 2015 and October 2018 and had been preoperatively tested by both PAT and MO performed on different days for 30 and 60 min. Near and distance deviations after 30 and 60 min of PAT and MO were compared to their baseline measurements obtained immediately after prism wear and before occlusion by alternate prism cover test. The near/distance measurements and required test duration to reveal the maximum deviation angle were also compared between PAT and MO.

**Results:**

Compared with the baseline, the near deviation by PAT significantly increased after 30 (*P* < 0.05) and 60 (*P* < 0.01) minutes but not the distance deviation. However, the increase after 30 min was not significant. By MO, neither near nor distance deviation showed a significant difference from the baseline after 30 and 60 min. PAT showed a significantly larger near deviation than MO at 30 and 60 min, but a larger distance deviation by PAT was only observed at 30 min.

**Conclusions:**

In patients with basic and convergence insufficiency types of IXT, a 30-minute PAT appears to be more effective than MO in revealing the maximum angle of deviation before strabismus surgery.

## Background

Intermittent exotropia (IXT) is an outward deviation that breaks down spontaneously into a manifest exotropia; and it has two states, exotropia and exophoria. With the ultimate goal to preserve patient’s good binocular vision and ability to maintain phoria, treatments for IXT include strabismus surgery, prism therapy, and orthoptics [1]. Although strabismus surgery is the common treatment modality especially for patients with large deviations, postoperative recurrence remains challenging [2].

The performed surgical procedure [3], a young age at surgery [2], presence of suppression [1], and an underestimated angle of deviation [4] have been described as the factors for postoperative recurrence. The angle of deviation is usually measured using the alternate prism cover test (APCT). Pritchard [4] and Kushner [5] have emphasized the significance of detecting the maximum deviation, which determines the required amount of surgery and leads to the prevention of postoperative recurrence. However, revealing the true deviation will be difficult if fusion cannot be sufficiently removed during APCT. We have experienced IXT patients with difficulty to break fusion in the tested eye possibly due to the surrounding atmosphere during the test. Besides, fusion may not be sufficiently removed depending on the skill of the examiner. The reported influences of tenacious proximal fusion (TPF) [6] (Scobee phenomenon [7]) and tenacious distance fusion [5] can also prevent IXT patients from revealing the true deviation during APCT. Therefore, other than APCT, a method that can measure the maximum angle of deviation before strabismus surgery is clinically essential to the management of IXT.

The prism adaptation test (PAT) and monocular occlusion (MO) are two effective methods to reveal the maximum angle of deviation [8, 9]. PAT reveals the maximum deviation using a prism that neutralizes the deviation, and MO brings out the underlying deviation by eliminating fusion with occlusion. Good surgical outcomes using surgical doses based on the angles of deviations measured after PAT and MO have been reported in patients with IXT [10–12]. While some studies concluded that PAT and MO are comparable in revealing the maximum deviation [13, 14], others reported the superiority of PAT over MO for obtaining a larger deviation [15–18] and no consensus has been reached. The debatable optimal test durations (time for prism wear and total occlusion time) for both methods to detect the maximum deviation vary among researchers [10, 11, 15, 16, 19–26], and this could be one of the reasons for the lack of consensus. Although a similar study has been conducted in esotropic patients [27], to our knowledge, no study has been conducted in IXT patients to investigate the optimal PAT and MO durations for revealing the maximum deviation.

This study aimed to compare the efficacies of preoperative PAT and MO for detecting the maximum angle of deviation within the same test duration (60 min) and to determine the optimal test durations for both methods.

## Methods

### Patients and the IXT types

Subjects in this retrospective study were 72 patients (34 males; age range, 6 to 76 years, mean, 19.0 ± 18.1 years) with IXT, who had received the initial strabismus surgery at the Kindai University Hospital between April 2015 and October 2018. Of 72, 54 (75 %) were under the age of 19. All the subjects had corrected visual acuity of 1.0 (LogMAR equivalent 0.0) or better and had received preoperative PAT and MO for 30 and 60 min. Exclusion criteria were patients with previous strabismus surgery, a vertical deviation angle of ≥ 5 prism diopter (PD), and a dissociated vertical deviation.

The subjects’ distance (5 m) and near (30 cm) deviations were measured by APCT with correction and their IXT types were classified as follows: basic type with a near/distance deviation difference of < 10 PD, convergence insufficiency type with a larger near deviation for more than 10 PD, and divergence excess type with a larger distance deviation for more than 10 PD. Of 72, 60 patients had basic IXT, 12 patients had convergence insufficiency type, and no patient had divergence excess type.

The protocol for this study was approved by the Ethics Committee of Kindai University Faculty of Medicine and adhered to the Declaration of Helsinki. Verbal informed consent was obtained from all the patients or parents.

### Test methods

The patient’s dominant eye was used for fixation in the test. To completely eliminate fusion, the examiner used a long cover when alternating the prisms to ensure that the eyes were not under binocular viewing. PAT used acrylic ophthalmic prisms (Gulden Ophthalmics, Pennsylvania, USA), and a prism with the distance deviation measured by APCT was applied over the patient’s non-dominant eye. The deviation angles immediately after prism wear (the baseline) and at 30 and 60 min were compared. In the MO test, the non-dominant eye was occluded, and the deviation angles before occlusion (the baseline) and 30 and 60 min after occlusion were compared. The measurements and the deviation changes after 30 and 60 min were also compared between PAT and MO. PAT and MO were performed on different days for each patient by the same examiner.

### Statistical analysis

Statistical analysis used the Steel-Dwass method to compare the angles after 30 and 60 min with the baseline. The Wilcoxon signed-rank test was used to compare the deviation changes between PAT and MO. A probability value of < 0.05 was considered statistically significant.

## Results

### Changes in the near and distance deviations after 30 and 60 min of PAT and MO

Table 1 shows the deviation angles measured at baseline and after 30 and 60 min of PAT and MO. The subjects’ distance and near deviations (mean ± standard deviation) by APCT were 26.4 ± 6.6 PD and 31.0 ± 6.8 PD, respectively. Compared with the baseline, the near deviation (mean ± SD) significantly increased after 30 (3.5 ± 3.9 PD, *P* < 0.05) and 60 min (4.1 ± 3.9 PD, *P* < 0.01) of PAT. However, the difference (0.6 ± 1.3 PD) between the measurements at 30 and 60 min was not significant. The distance deviations at 30 and 60 min did not significantly differ from the baseline. Compared to the baseline, neither near nor distance deviation significantly increased after 30 and 60 min of MO.

**Table 1:** The PAT and MO measurements of deviations

		Baseline	30 min	60 min
PAT	near	30.9 ± 6.9	34.4 ± 6.7	35.0 ± 6.7
	distance	26.4 ± 6.6	28.6 ± 7.6	29.0 ± 7.6
MO	near	31.0 ± 6.8	32.3 ± 6.7	32.8 ± 6.8
	distance	26.4 ± 6.6	27.8 ± 7.0	28.3 ± 7.1

Values are presented as average ± standard deviation (PD)

**Fig. 1:**
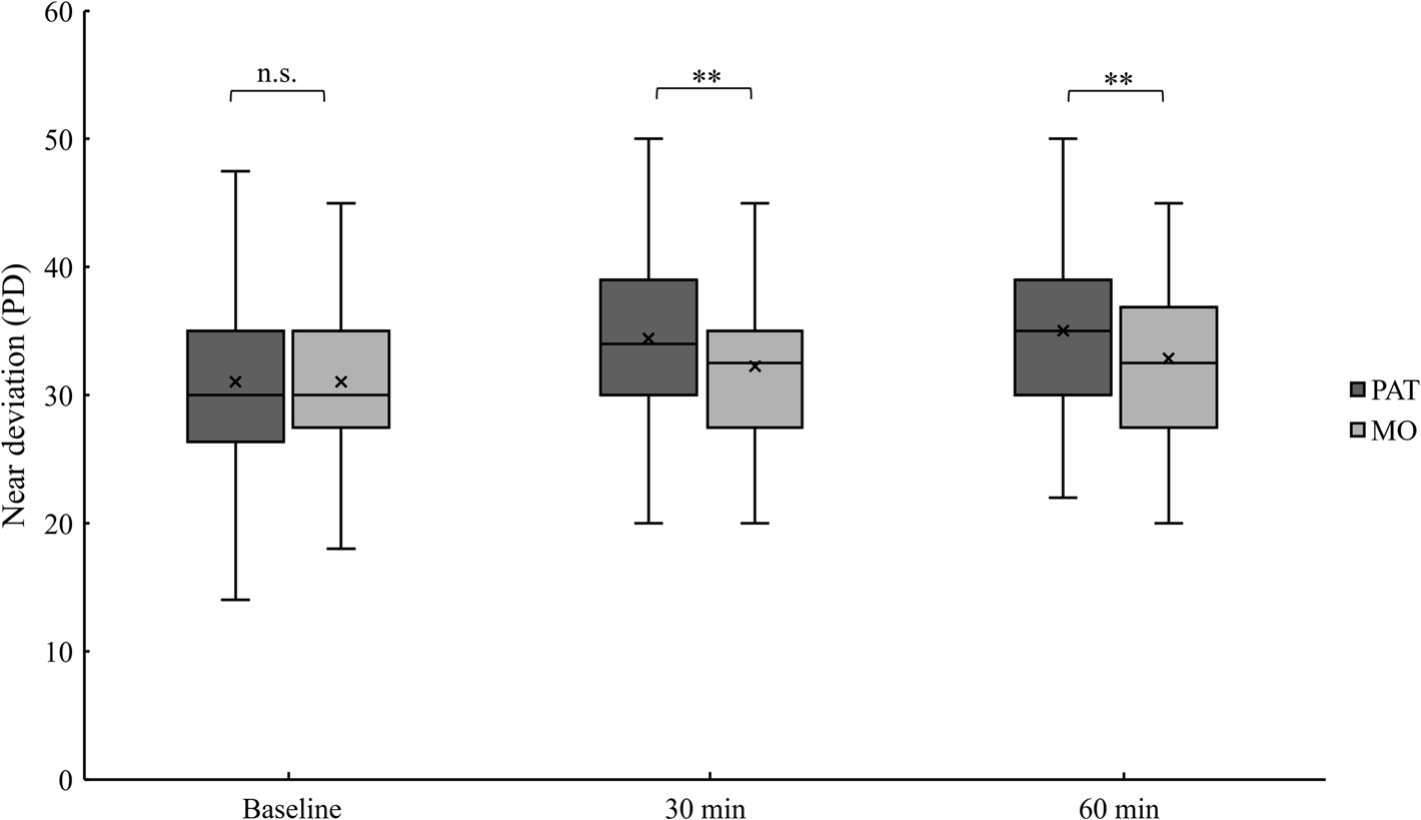
Comparison of the near (30 cm) deviations by PAT and MO at the three time points. While the baseline APCT measurements did not significantly differ, the PAT deviations at 30 and 60 min were significantly larger than those by MO. Analysis used the Wilcoxon signed-rank test (**P < 0.01; n.s.: not significant)

**Fig. 2:**
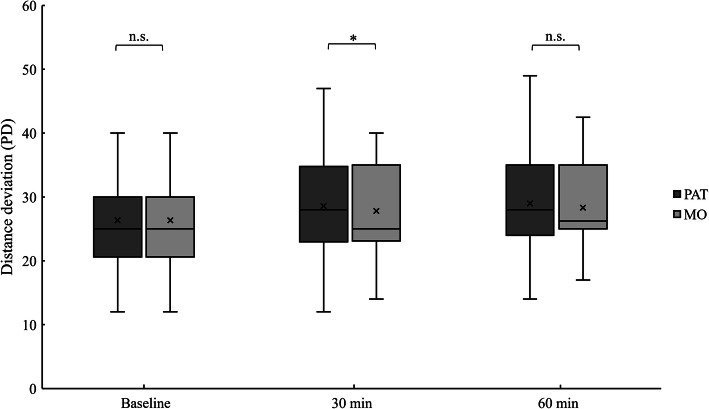
Comparison of the distance (5 m) deviations by PAT and MO at the three time points. The distance deviations by PAT were also larger than those by MO, but the differences were not as distinct as the differences seen in the near deviation. Analysis used the Wilcoxon signed-rank test (*P < 0.05; n.s.: not significant)

The near deviations by PAT at 30 and 60 min were significantly larger than those by MO (*P* < 0.01, Wilcoxon signed-rank test; Fig. 1). PAT also showed a significantly larger distance deviation than MO at 30 min (*P* < 0.05, Wilcoxon signed-rank test; Fig. 2) but not at 60 min.

### Changes of patients’ IXT types

After PAT, 9 (15 %) of the 60 patients with basic IXT converted to convergence insufficiency type. In these patients, the near/distance deviation differences by APCT and PAT were 5.6 ± 1.0 PD and 10.4 ± 0.9 PD, respectively. The 12 patients with convergence insufficiency type before PAT remained the same after PAT.

## Discussion

With a 60-minute test duration for PAT and MO, the angles of deviation measured after PAT were larger than those measured after MO. Furthermore, significant differences in near and distance deviations were only observed at 30 min and no significant difference between both methods was seen after 30 min. These results demonstrated the efficacy of a 30-minute preoperative PAT and suggested that the stabilized fusion by prism adaptation (PA) could be more effective than the eliminated fusion by MO for revealing the maximum angle of deviation in IXT.

The current result showed that the angles of exodeviation measured after PAT were significantly larger than those after MO and this was in agreement with previous results [15–18]. The PAT and MO measurements may be comparable, but a larger angle of deviation after MO has not been reported previously. These results suggest that the preoperative use of PAT could be more effective than MO in revealing the maximum angle of deviation in IXT. A similar study in 19 patients with normosensoric esotropia concluded that it appears advisable to tailor surgery to patient’s squint angle after PA [27]. In patients with IXT, motor fusion during PA is suppressed and sensory fusion stability is sustained in order for the displaced retinal image to be fused on the two foveae. Unlike MO that eliminates fusion with occlusion, PA brings sensory fusion into a stable status under binocular viewing and thus better elicits the underlying deviation.

The PAT measurements at near showed a greater increase than the measurements at distance in this study. A previous study has reported a similar observation [19]. Furthermore, 9 patients with basic IXT before PAT converted to convergence insufficiency type after their near deviations increased. Vergence aftereffect (or slow vergence) is a temporary change in the eye position resulted from sustained fusional convergence. Reportedly, vergence aftereffect could mask the true near deviation in IXT and cause underestimated near deviation [26, 28–30]. In addition, proximal convergence and vergence aftereffect also cause a higher accommodation convergence / accommodation (AC / A) ratio in IXT [31]. On the other hand, a smaller AC/A ratio is observed after PAT [15]. With PAT showing the maximum angle of near deviation in this study, we therefore suspected that PA might have eliminated the influences of proximal convergence and vergence aftereffect and elicited the true underlying near deviation.

The distance and near deviations after 30 and 60 min of MO did not significantly differ from the baseline measurements by APCT, and they were all smaller than those by PAT. Hans and colleagues reported that the average maximum angles of deviation at distance and near on 3 or more examinations before MO are larger than those measured after 1-hour MO [32]. This suggests that 1-hour MO might not be able to reveal the same amount of deviation as 1-hour PAT. Furthermore, since MO and APCT shared the same mechanism that eliminated fusion by occlusion, the eliminated fusion might not be sufficient and this also explained why the maximum deviations by MO were smaller than those by PAT in this study.

Previously reported optimal PA time ranges from 30 min to 2 weeks [10, 11, 15, 17–19] and no consensus has been reached. A shorter PA time is always desirable especially in pediatric patients. In this study, near deviations after 30 and 60 min of PAT were significantly different from the baseline, but the increase after 30 min was not significant. In addition, no significant difference in near and distance deviations was observed after 30 min between PAT and MO. These results suggest the potential of a 30-minute PA for revealing the underlying deviation.

This study has some limitations. Because this study did not include patients with either divergence excess or pseudo-divergence excess IXT, the current results could only apply to patients with basic or convergence insufficiency type of IXT. The occlusion and PA durations were set for 30 and 60 min and thus, it was not clear whether and how the angle of deviation would further change beyond 60 min. However, no subjects showed an increase of ≥ 5 PD between 30 and 60 min; and the measurements at 30 and 60 min did not significantly differ. We therefore considered that the deviation was unlikely to significantly increase after 60 min. Because the AC/A ratios before and after MO and PAT were not available, the association between the AC/A ratio and the increase in the near deviation could not be examined.

In conclusion, PAT is more useful than MO for preoperative detection of the maximum deviation in IXT. No significant changes in the near and distance deviations were observed after 30 min of PAT. Therefore, a 30-minute PAT has the potential to reveal the true underlying deviation in patients with basic and convergence insufficiency types of IXT.

## Data Availability

The datasets used and analyzed in the current study are available from the corresponding author on reasonable request.

## Abbreviations

IXT: Intermittent exotropia
PAT: Prism adaptation test
MO: Monocular occlusion
APCT: Alternate prism cover test
PD: Prism diopter
PA: Prism adaptation
AC/A: Accommodation convergence / accommodation
